# Inappropriate Expression of PD-1 and CTLA-4 Checkpoints in Myeloma Patients Is More Pronounced at Diagnosis: Implications for Time to Progression and Response to Therapeutic Checkpoint Inhibitors

**DOI:** 10.3390/ijms24065730

**Published:** 2023-03-17

**Authors:** Anna Kulikowska de Nałęcz, Lidia Ciszak, Lidia Usnarska-Zubkiewicz, Edyta Pawlak, Irena Frydecka, Magdalena Szmyrka, Agata Kosmaczewska

**Affiliations:** 1 Hematology and Hematological Oncology Department, Provincial Hospital, 45-061 Opole, Poland; 2 Hirszfeld Institute of Immunology and Experimental Therapy, Polish Academy of Sciences, 53-114 Wroclaw, Poland; 3 Department of Hematology, Blood Neoplasms, and Bone Marrow Transplantation, Wroclaw Medical University, 50-367 Wroclaw, Poland; 4 Department of Rheumatology and Internal Diseases, Wroclaw Medical University, 50-556 Wroclaw, Poland

**Keywords:** multiple myeloma, PD-1, CTLA-4, immune checkpoint inhibitors, time to progression, clinical response

## Abstract

Multiple myeloma (MM) is a hematologic malignancy characterized by severely profound immune dysfunction. Therefore, the efficacy of drugs targeting the immune environments, such as immune checkpoint inhibitors (ICIs), is of high clinical importance. However, several clinical trials evaluating ICIs in MM in different therapeutic combinations revealed underwhelming results showing a lack of clinical efficacy and excessive side effects. The underlying mechanisms of resistance to ICIs observed in the majority of MM patients are still under investigation. Recently, we demonstrated that inappropriate expression of PD-1 and CTLA-4 on CD4 T cells in active MM is associated with adverse clinical outcomes and treatment status. The aim of the current study was to determine the usefulness of immune checkpoint expression assessment as a predictive biomarker of the response to therapeutic inhibitors. For this purpose, along with checkpoint expression estimated by flow cytometry, we evaluated the time to progression (TTP) of MM patients at different clinical stages (disease diagnosis and relapse) depending on the checkpoint expression level; the cut-off point (dividing patients into low and high expressors) was selected based on the median value. Herein, we confirmed the defective levels of regulatory PD-1, CTLA-4 receptors, and the CD69 marker activation in newly diagnosed (ND) patients, whereas relapsed/refractory patients (RR) exhibited their recovered values and reactivity. Additionally, substantially higher populations of senescent CD4^+^CD28^−^ T cells were found in MM, primarily in NDMM subjects. These observations suggest the existence of two dysfunctional states in MM CD4 T cells with the predominance of immunosenescence at disease diagnosis and exhaustion at relapse, thus implying different responsiveness to the external receptor blockade depending on the disease stage. Furthermore, we found that lower CTLA-4 levels in NDMM patients or higher PD-1 expression in RRMM patients may predict early relapse. In conclusion, our study clearly showed that the checkpoint level in CD4 T cells may significantly affect the time to MM progression concerning the treatment status. Therefore, when considering novel therapies and potent combinations, it should be taken into account that blocking PD-1 rather than CTLA-4 might be a beneficial form of immunotherapy for only a proportion of RRMM patients.

## 1. Introduction

Despite the current advances in treatment seen with the inclusion of proteosome inhibitors (PIs), immunomodulatory drugs (IMIDs), and chimeric T cell therapy (CAR-T) multiple myeloma (MM) remains an almost universally incurable malignancy. Along with reducing the bulk of myeloma cells by conventional therapy, host factors including the cytotoxic capacity of activated T cells is essential for tumor eradication and clinical outcomes of MM therapy. Accumulating evidence has shown that tumor-induced immune dysfunction in MM patients seems to be greater than in other B cell malignancies [1,2,3], and includes dysfunction of dendritic and T cells [4], accumulation of suppressive cell types [2,4], cytotoxic T cell/Treg and Th17/Treg imbalance [5,6], and T cell hyporesponsiveness [7,8].

The dysfunction of T cells in cancer patients is characterized by anergy, senescence, and/or exhaustion [9,10,11], namely the states sharing expression of the multiple inhibitory molecules implicated in the impaired effector functions and hyporesponsiveness. Overcoming the hyporesponsiveness of T cells could reinvigorate the host’s immune response and restore antitumor immunity. Available data, including ours, demonstrates that T cells in patients with MM display features of exhaustion and senescence [6,12]. It is interesting to note that both dysfunctional states may exist at the same disease stage. Determination of the extent of senescence and/or exhaustion within the T cell compartment remain confusing due to several similarities. While both states share overlapping functional and phenotypic features including the expression of regulatory receptors (e.g., PD-1 and CTLA-4), increased cell cycle arrest, and affected effector functions, they also have distinct regulatory and molecular mechanisms controlling their development and disturbed antitumor functions [13,14,15]. Another difference is the potential for reversion as a consequence of modulation of regulatory pathways after external receptor blockade, a feature attributed only to exhaustion [13,16]. An increasing body of evidence suggests that the extent of the clinical response to checkpoint inhibitors might be closely related to the level of checkpoint expression on T cells [6,16]. Therefore, the identification of dysfunctional states predominating in MM patients regarding the clinical stage could help determine if T cell hyporesponsiveness is reversible by manipulating the immune checkpoint blockade, thus predicting the clinical response to this therapeutic approach.

Accordingly, our recent study was conducted to assess the usefulness of immune checkpoints as predictive biomarkers of the response to therapeutic checkpoint inhibitors at different clinical stages, namely NDMM and RRMM patients [6]. In the current study, we confirmed the suboptimal level of CTLA-4 and PD-1 in circulating MM CD4 T cells, primarily in NDMM patients. Furthermore, we observed that the level of checkpoint expression may predict the clinical outcome, when considering TTP, in relation to the treatment status; lower CTLA-4 expression at disease diagnosis or higher PD-1 levels in RRMM patients might predict early relapse. Our results clearly indicate that blocking immune checkpoints might be a beneficial form of immunotherapy for only a proportion of RRMM patients.

## 2. Results

### 2.1. Circulating Myeloma CD4 T Cells Contain Lower Levels of Immune Checkpoints at Diagnosis

As recent clinical trials of the administration of immune checkpoint inhibitors (ICIs) in MM showed real disappointment [16,17,18,19,20], we wanted to verify whether the onset and/or exacerbation of MM is accompanied by alterations in the expression of immune checkpoints, thereby affecting their usefulness as targets for therapeutic inhibitors. Therefore, we assessed PD-1 and CTLA-4 expression in the peripheral blood (PB) CD4^+^ T cell subsets involved in the antitumor response in MM patients at disease diagnosis and relapse.

**Figure 1:**
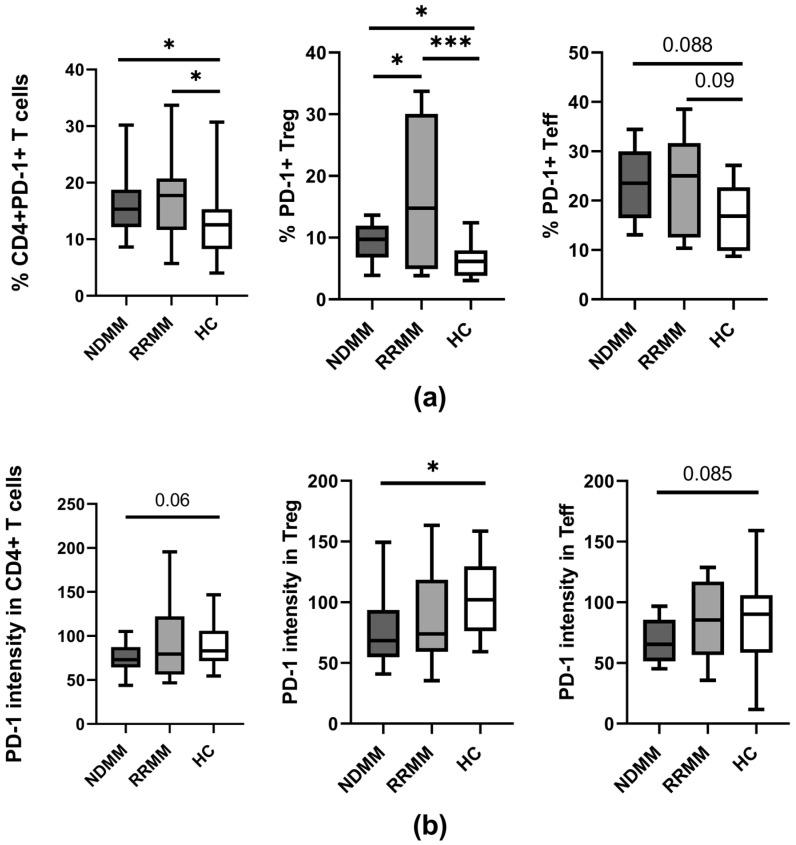
PD-1 expression on PB CD4 T cell subtypes of MM patients in the different clinical stages and healthy controls. (**a**) Frequency of PD-1-expressing CD4^+^ T cells (identified as CD3^+^CD4^+^), Treg (identified as CD4^+^CD127^−^), and Teff (identified as CD4^+^CD127^+^) cells in healthy controls (HC) (n = 20) and patient subgroups (NDMM and RRMM) (n = 26 and n = 14, respectively). (**b**) PD-1 level (determined as mean fluorescence intensity) in CD4^+^ T cells, Treg, and Teff cells in HC (n = 20), NDMM (n = 26), and RRMM (n = 14) patients. Boxes and whiskers show 25th and 75th interquartile range and minimum–maximum, respectively; the median is the central line in each box. The differences between the studied groups were statistically evaluated using Kruskal–Wallis, ANOVA, and Mann–Whitney U tests. * *p* < 0.05, *** *p* < 0.001.

In our study, as is shown in Figure 1a, a median proportion of CD4^+^ T cells expressing the PD-1 checkpoint was found to increase in all MM patients regardless of disease stage when compared to controls (*p* < 0.05). While an expansion of PD-1^+^ Teff cells was similar in all patients, Treg cells from RRMM patients expressed the PD-1 molecule on a significantly higher proportion of cells than in the NDMM group (*p* = 0.037) (Figure 1a).

Remarkably, a quantitative analysis of PD-1 expression on PB CD4^+^ T cells showed lower levels in NDMM patients compared to healthy controls (*p* = 0.06) (Figure 1b). While a decline of PD-1 was observed in the whole population of NDMM CD4^+^ T cells, including both Teff and Treg subsets, the loss of PD-1 was more pronounced in Treg cells (*p* = 0.016) (Figure 1b); in Teff cells, a decrease of PD-1 was of borderline significance (*p* = 0.08) (Figure 1b). In RRMM patients, PD-1 expression was also down-regulated primarily in the Treg subset; however, its median values were statistically comparable to those observed in corresponding healthy cells (Figure 1b). Although some differences in PD-1 expression between patient groups were observed, they did not reach statistical significance.

**Figure 2:**
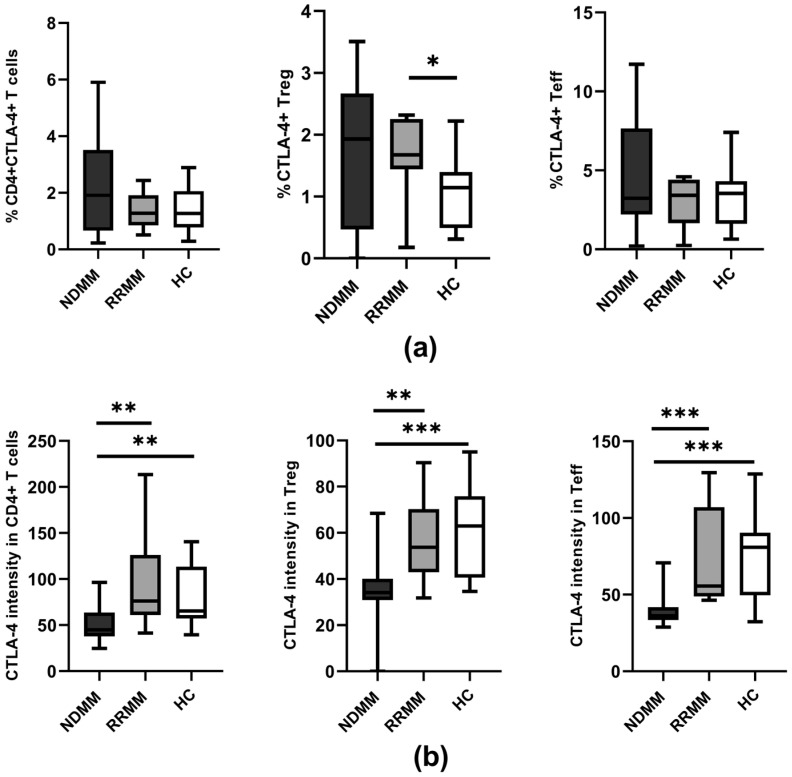
CTLA-4 expression on PB CD4 T cell subtypes of MM patients in the different clinical stages and healthy controls. (**a**) Frequency of CTLA-4 expressing CD4^+^ T cells (identified as CD3^+^CD4^+^), Treg (identified as CD4^+^CD127^−^), and Teff (identified as CD4^+^CD127^+^) cells in healthy controls (HC) (n = 20) and patient subgroups (NDMM and RRMM) (n = 26 and n = 14, respectively). (**b**) CTLA-4 level (determined as mean fluorescence intensity) in CD4^+^ T cells, Treg, and Teff cells in HC (n = 20), NDMM (n = 26), and RRMM (n = 14) patients. Boxes and whiskers show 25th and 75th interquartile range and minimum–maximum, respectively; the median is the central line in each box. The differences between the studied groups were statistically evaluated using Kruskal–Wallis, ANOVA, and Mann–Whitney U tests. * *p* < 0.05, ** *p* < 0.01, *** *p* < 0.001.

As is demonstrated in Figure 2a, we also found no significant differences in the proportion of CTLA-4 expressing cells within the examined subsets between the participants studied, except for the higher abundance of CTLA-4^+^ Treg cells in RRMM patients when compared to healthy controls (*p* = 0.031). Remarkably, similarly to PD-1 expression, a quantitative estimation of CTLA-4 showed that the only group exhibiting a markedly down-regulated level of CTLA-4 on CD4 T cells, including Teff and Treg cells, was NDMM patients (*p* ≤ 0.008 and *p* ≤ 0.005, respectively) compared to normal levels in corresponding cells from the RRMM and healthy groups (Figure 2b). Additionally, there were no differences in CTLA-4 expression between RRMM patients and controls regarding the studied CD4^+^ T cell subsets (Figure 2b).

From our results it seems that the lower levels of both immune checkpoints in MM CD4 T cells suggest they are not appropriate targets for therapeutic inhibitors in MM, at least at disease onset, and, consequently, should not to be considered in first-line treatment settings. Furthermore, chemo- and/or immunotherapy of MM, despite the risk of the development of refractoriness, is capable of reinforcing both PD-1 and CTLA-4 checkpoint expression, making them more attainable to the therapeutic inhibitors in RRMM.

### 2.2. Lower CTLA-4 Levels at Myeloma Diagnosis Predispose to a Shortened TTP

**Figure 3:**
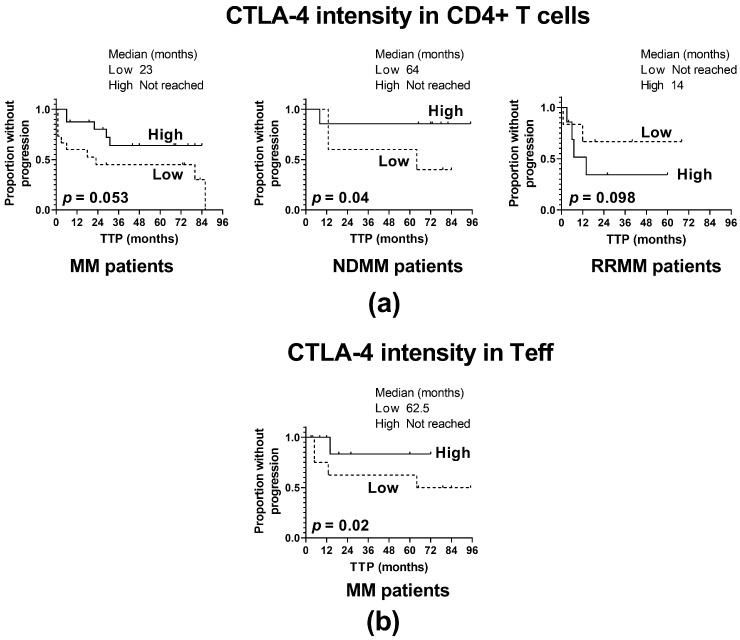
Influence of CTLA-4 expression on the time to progression (TTP). (**a**) TTP in patients with high and low CTLA-4 expression in CD4^+^ T cells (shown as CTLA-4 fluorescence intensity) identified as >median and ≤median values, respectively, in the whole patient cohort (MM) (n = 40) and patient subgroups (NDMM and RRMM) (n = 26 and n = 14, respectively). (**b**) TTP in patients with high and low CTLA-4 expression in Teff cells (shown as CTLA-4 fluorescence intensity) identified as >median and ≤median values, respectively, in the whole patient cohort (MM) (n = 40). The log-rank test was performed for the Kaplan–Meier curves.

Next, we analyzed whether the observed alterations in PD-1 and CTLA-4 expression may influence the clinical outcome of MM. Therefore, we assessed the TTP of MM patients at the different clinical stages (disease onset and relapse/refractoriness) depending on the expression of both immune checkpoints on CD4 T cells. The cut-off point was selected based on the median value of the checkpoint expression (measured at qualitative and quantitative levels). The statistically significant associations are shown in Figure 3.

For the whole MM patient cohort, regardless of CD4^+^ T cell subtypes, a weak positive association of CTLA-4 level on CD4^+^ T cells with TTP was indicated (*p* = 0.053) (Figure 3a); however, this relationship was found to be strengthened only with regards to the Teff subset (*p* = 0.02) (Figure 3b), and CTLA-4 expression on Treg was not associated with TTP. Furthermore, clinical subanalysis revealed that the above association might be assigned to NDMM patients (Figure 3a); patients with a CTLA-4 level below the median value had significantly shorter TTP than those with a higher CTLA-4 expression (*p* = 0.04). In contrast, RRMM patients exhibited no statistically significant association of CTLA-4 fluorescence intensity with TTP (*p* = 0.098) (Figure 3a). Likewise, no impact was found of changes in CTLA-4 distribution within CD4^+^ T cells on MM progression in either group of patients.

Our study demonstrating the association of CTLA-4 loss in CD4 T cells with shortened TTP seen in NDMM patients points to the possibility that blockade of CTLA-4 in MM may be an unfavorable strategy at diagnosis.

### 2.3. Higher PD-1 Expression May Predict Early Relapse in RRMM Patients

Given it is noted that MM patients at diagnosis exhibited an impaired PD-1 level, we next examined its association with clinical outcomes in terms of TTP as well.

**Figure 4:**
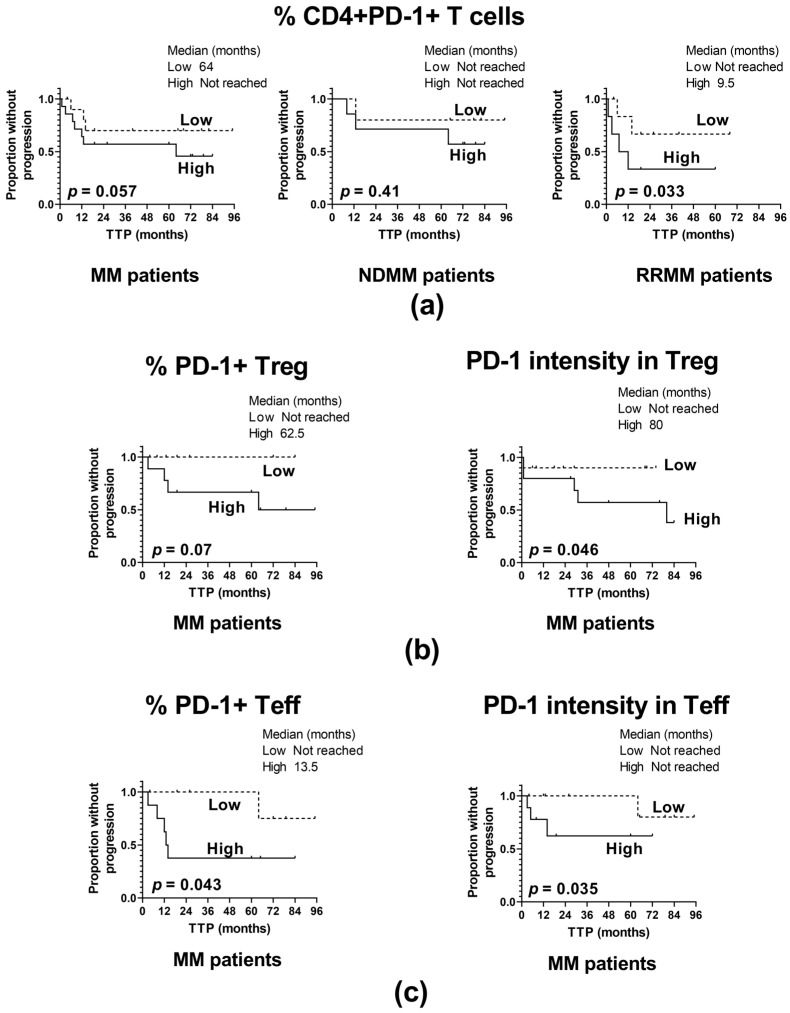
Influence of PD-1 expression on the time to progression (TTP). (**a**) TTP in patients with high and low frequency of PD-1 positive CD4^+^ T cells identified as >median and ≤median values, respectively, in the whole patient cohort (MM) (n = 40) and patient subgroups (NDMM and RRMM) (n = 26 and n = 14, respectively). (**b**) TTP in patients with high and low PD-1 expression in Treg cells (shown as the frequency of PD-1^+^ Treg cells and as PD-1 fluorescence intensity in Treg), identified as >median and ≤median values, respectively, in the whole patient cohort (MM) (n = 40). (**c**) TTP in patients with high and low expression of PD-1 in Teff cells (shown as frequency of PD-1^+^ Teff and as PD-1 fluorescence intensity in Teff) identified as >median and ≤median values, respectively, in the whole patient cohort (MM) (n = 40). The log-rank test was performed for the Kaplan–Meier curves.

For the whole patient cohort, as is demonstrated in Figure 4, several inverse associations of TTP with the frequency of PD-1-expressing CD4^+^ T cells (*p* = 0.057) (Figure 4a) have been found: PD-1^+^ Treg cells (*p* = 0.07) (Figure 4b), PD-1^+^ Teff (*p* = 0.043) (Figure 4c), and the level of PD-1 in Treg and Teff cells (*p* = 0.046 and *p* = 0.035, respectively) (Figure 4b and 4c, respectively). Together these indicate that high-PD-1 expressors experienced an MM relapse significantly earlier than those with PD-1 expression below the median values. However, patient analysis based on the clinical division according to MM stage revealed that the above observation was primarily assigned to the RRMM group, where patients with percentages of CD4^+^PD-1^+^ T cells over the median value had markedly shorter TTP compared to those with a lower frequency of these cells (*p* = 0.033) (Figure 4a); of note, such an association was not shown for NDMM patients (*p* = 0.41) (Figure 4a).

From the above data it appears that the administration of PD-1 inhibitors might be a beneficial form of therapy for a proportion of RRMM patients, particularly those exhibiting higher PD-1 expression within CD4^+^ T cells.

### 2.4. CD4 T Cells from NDMM Patients Retain In Vivo Lower Reactivity to Stimuli

Having demonstrated that the dysregulated expression of immune checkpoints on T cells may be a consequence of altered in vivo stimulation, we also analyzed the systemic activation of CD4 T cells in all individuals studied. The assessment of T cell reactivity is important in predicting the biological effectiveness of the checkpoint blockade [13,21].

**Figure 5:**
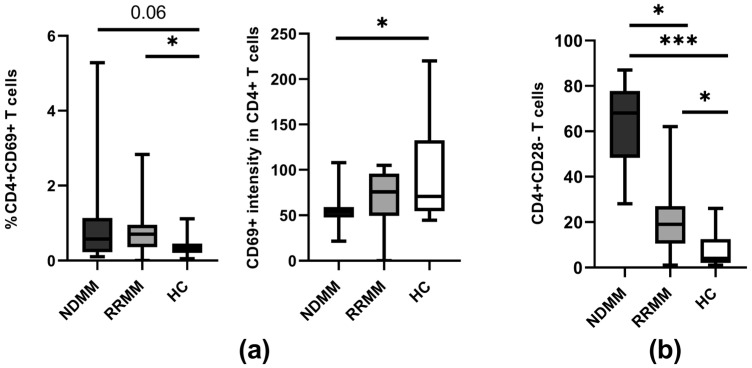
Markers of CD4 T cell reactivity. (**a**) Expression of the CD69 activation marker on CD4^+^ T cells in PB of healthy controls (HC) (n = 20) and patient subgroups (NDMM and RRMM) (n = 26 and n = 14, respectively). (**b**) Frequency of CD28 lacking CD4^+^ T cells at the different MM stages. Boxes and whiskers show 25th and 75th interquartile range and minimum–maximum, respectively; the median is the central line in each box. The differences between the studied groups were statistically evaluated using Kruskal–Wallis, ANOVA, and Mann–Whitney U tests. * *p* < 0.05, *** *p* < 0.001.

While we noted an increased proportion of CD4^+^CD69^+^ T cells in the PB of all patients (*p* < 0.06), statistically significant differences were found only between the RRMM group and healthy controls (*p* = 0.027) (Figure 5a). Remarkably, the median fluorescence intensity of the activation marker CD69 was the lowest in the CD4^+^ T cells from NDMM patients and it significantly differed from that obtained in corresponding healthy cells (*p* = 0.017). Although the difference in CD69 expression was also observed in comparison to the RRMM group, it remained at a statistically similar level (Figure 5a).

The above results indicate that the level of immune checkpoint expression in MM corresponds with the suboptimal activation of T cells. Therefore, inappropriate expression of checkpoints may not be responsible for MM T cell hyporesponsiveness observed primarily at disease onset.

### 2.5. CD28 Loss Related to Cell Senescence Is More Pronounced at Myeloma Diagnosis

Despite the altered pattern of immune checkpoint expression, the lack of CD28 could also help to identify a predominant dysfunctional state in MM CD4 T cells related to immune suppression. Given that it has been demonstrated that the lack of CD28 is one of the constant features of senescent T cells, CD28 negativity has been proposed as a surrogate senescence marker of T cells [13,22]. Therefore, we analyzed the frequency of pathogenic PB CD4^+^CD28^−^ T cells at different MM stages in order to predict a clinical response to ICIs.

We found a substantial increase in the proportion of CD4^+^CD28^−^ T cells in circulation among all MM patients compared to the controls (*p* < 0.0001). However, detailed analysis revealed a significant difference in the abundance of these pathogenic cells among patients regarding disease stage, with the predominance of senescent CD4^+^CD28^−^ T cells evident in the NDMM group when compared to RRMM patients (*p* < 0.001) (Figure 5b).

Our data clearly showed that MM immunopathology is characterized by a higher frequency of CD4^+^CD28^−^ T cells, which is more pronounced in NDMM patients. This may clearly indicate prevalence of the senescent state in MM and points toward the suggestion that reversion of hyporesponsiveness by administrating therapeutic checkpoint inhibitors in MM could be associated with an increasing risk of ineffectiveness.

### 2.6. The Significance of Clinico-Pathological Features in Prediction of Myeloma Early Relapse

**Figure 6:**
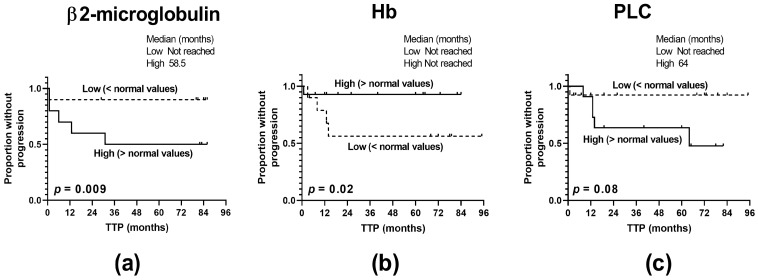
Predictive significance of the clinico-pathological characteristics of MM patients. (**a**) Levels of β2-microblobulin, (**b**) anemia, and (**c**) percentages of circulating plasmacytes (PLCs) affect the time to progression (TTP) of MM patients (n = 40). The log-rank test was performed for the Kaplan–Meier curves.

In order to determine the representativeness of our cohort of MM patients, we assessed the impact of the clinico-pathological features of patients on the MM clinical outcome regarding the time to disease relapse. Among the clinical characteristics studied, including patient age, the International Staging System (ISS), anemia, and the levels of β2-microglobulin, albumin, creatinine, plasmacytes, lactate dehydrogenase (LDH), and serum calcium, we found that hyper-β2-microglobulinemia, anemia, and the higher abundance of circulating plasmacytes shortened the time to MM progression (*p* = 0.009, *p* = 0.02, and *p* = 0.08, respectively), thus indicating their predictive significance and enrollment of suitable patients (Figure 6).

## 3. Discussion

The reversal of T cell hyporesponsiveness plays an essential role in myeloma immunotherapy aimed at the restoration of the host’s immune response. Growing evidence has shown that defective tumor immunity contributes to compromised effectiveness of anti-myeloma therapy applied so far mainly in advanced high-risk RRMM [23]. The available research demonstrated that MM T cells, depending on the clinical stage, may display features of immune senescence and/or functional exhaustion [6,12,24]. In order to resolve the mechanism responsible for the hyporesponsiveness of MM T cells, it is crucial to distinguish between both dysfunctional states to achieve their adequate and effective control with the appropriate therapeutic approaches.

Herein, we report that in patients with active MM the alterations of the immune checkpoint level depend on the clinical stage of the disease associated with treatment status, and correspond to the extent of the systemic activation of circulating CD4 T cells. In particular, defected levels of PD-1 and CTLA-4 checkpoints, as well as CD69 were observed in MM primarily at diagnosis. Furthermore, we noted that patients, although relapsed, also exhibited inappropriate checkpoint expression, thus confirming the recent suggestion that immune checkpoints are not responsible for T cell hyporesponsiveness in MM [6,24]. The above observation points to the possibility that immune checkpoints might not be appropriate targets for therapeutic inhibitors in MM, and indicates that T cell-related immune suppression in MM cannot be effectively reversed by manipulating extrinsic regulatory pathways. Remarkably, our observation on an association of the lower CTLA-4 expression on CD4 T cells with shortened TTP in NDMM patients confirms the suggestion that blocking CTLA-4 in MM may be an unfavorable therapeutic strategy for a proportion of MM patients, primarily at diagnosis. This finding is consistent with former observations, including ours, on the impact of genetic variations of the *CTLA-4* gene involved in lower CTLA-4 protein expression with increasing susceptibility to MM development [25,26,27]. Similar dependence of the response to a CTLA-4 blockade on the level of CTLA-4 expression was previously observed in chronic lymphocytic leukemia by our group [28].

Moreover, inappropriate levels of PD-1 and CTLA-4 in MM CD4 T cells together with the increased subset of CD4^+^CD28^−^ T cells found in our patient cohort have been demonstrated as features of senescent T cells, thus indicating senescence as a predominant dysfunctional state in MM [24]. Our observation is in accordance with a study by Zeller-Riese et al. [12], who recently found that T cells from MM patients express several molecules associated with either T cell exhaustion (e.g., PD-1 and CTLA-4) or T cell senescence (a lack of CD28). They also reported, however, that the enhancement of the senescent CD28-negative T cell population was more pronounced in NDMM patients in comparison to RRMM subjects. These results clearly correspond with our recent study performed in the same patient cohort [6], demonstrating the expansion of CD4 T cells exhibiting a senescence-associated secretory effector phenotype (SASP) [29], primarily in NDMM patients. This is suggestive of the significance of immune senescence in myeloma development, rather than anergy as a dominant functional state of hyporesponsiveness, and emphasizes a role for determining the extent of T cell senescence [6]. Remarkably, the data available have shown that T cell senescence is caused by intrinsic signals induced by DNA damage and can be reversed pharmacologically, but not with an external receptor blockade [13,22,30,31], thereby minimizing the role for checkpoint inhibitors in MM immunotherapy. It should be emphasized, however, that T cell clones in MM have recently been demonstrated not to be related to shortened telomeres, which may imply that their senescence is potentially reversible in MM, if the underlying mechanism is elucidated [24]. Nonetheless, estimation of the senescent T cell population in MM may be of clinical relevance in terms of predicting the clinical response to therapeutic checkpoint inhibitors.

The defective levels of immune checkpoints observed in the present study, more pronounced in the Treg subset, may also imply the inappropriate prevention of autoimmunity [32]. Likewise, in the same patient cohort, we recently found a higher population of CD4 T cells with capacity for inflammatory IL-17 and IFN-γ cytokine secretion with an increased Th17/Treg ratio when compared to the values obtained in the RRMM group [6]. Although the Treg cell population was found to be expanded in all studied patients, the current analysis revealed that this subset is mostly affected by impaired expression levels of PD-1 and CTLA-4 at disease diagnosis, which might compromise the Treg regulatory function. Given that checkpoint expression in Treg cells is associated with suppressive activity [33,34,35], the decreased level of checkpoints in the Treg population is suggestive of the shift in the immune balance toward autoimmune inflammatory conditions, especially in NDMM patients. In fact, the augmented population of CD4^+^CD28^−^ T cells that we found in all MM patients was previously shown to secrete inflammatory cytokines, which corresponds with the phenotypic and functional characteristics of autoreactive cytotoxic CD4^+^ T cells [6,36,37,38]. The notion that among MM patients, the CD4^+^CD28^−^ T cell population is significantly enriched, primarily at disease onset, is in line with the current phenotypic analysis of Tregs and strongly indicates the possibility of a higher incidence of autoimmune responses in MM. In fact, a personal history of autoimmune disease was found to be associated with a significantly higher risk of the monoclonal gammopathy of undetermined significance (MGUS) and MM, indicating a common genetic susceptibility between autoimmunity and plasma cell disorders [39,40]. It has also been demonstrated that co-morbidity of the autoimmune disease might be prognostic of a worse survival rate in MM patients [39], and suboptimal immune checkpoint expression cannot be excluded when considering the underlying mechanism. Our finding that the lower CTLA-4 expression on CD4 T cells may predict early relapse in NDMM patients also seems to confirm these associations. Therefore, an increasing risk of exaggeration of autoimmune events should be taken into account when considering blocking immune checkpoints in MM [41]. A case of an NDMM patient who developed a lethal immune-related myocarditis after a single dose of pembrolizumab (a PD1-L inhibitor) in combined therapy with lenalidomide and dexamethasone seems to strengthen the above concern, and indicates that therapeutic checkpoint inhibitors should be administered in MM with extreme caution, if at all, especially in first-line treatments [42].

Interestingly, our present study also showed that chemo- and/or immunotherapy of MM, despite the well-known risk of the development of refractoriness [43], is capable of reinforcing both PD-1 and CTLA-4 checkpoint expression, and this notion is consistent with other studies [6,12,44]. The post-treatment ligation of regulatory receptors resulting in the increase and recovery of T cells found in this study has previously been demonstrated as a key phenotypic feature of cell exhaustion, which is also a dysfunctional state related to MM T cell hyporesponsiveness [12]. It should be emphasized, however, that unlike the cell senescence predominant in NDMM patients, the exhaustion of T cells observed mainly in relapse has been shown to be reversible upon inhibitory receptor blockade [13,23]. Here we report that an increase in immune checkpoint expression seen in RRMM patients and accompanied by CD69 reversion is suggestive of the treatment-related tendency to the restoration of reactivity in CD4 T cells, thus making them more attainable for therapeutic inhibitors in RRMM. Of note, exhaustion has been proposed as a mechanism of the prevention of T cell loss and the retention of T cell clones required for immune surveillance and tumor immunity [13]. In fact, expanded clones of cytotoxic T cells exist in patients with MM and other hematologic malignancies [35,45,46,47,48,49,50,51]. Although exhausted T cells are hyporesponsive in vitro, their presence in the blood of MM patients is related to better prognosis, probably due to their potential to reverse cell dysfunction [45,46,52,53]. However, our current study, showing that high-PD-1 expressors (primarily within the Teff population) in RRMM patients exhibit a shortened TTP, is indicative of the possibility that a PD-1 blockade might be a beneficial form of immunotherapy for a subset of RRMM patients depending on their PD-1 expression level. This notion is in line with a recent study by Alrasheed et al. [54], who demonstrated that a high level of PD-1^+^ Teff cells predicts early progression in MM. In our recent study [6], we consistently found an association of high PD-1 expression in Teff and Treg cells in MM with an adverse clinical outcome. Genetic susceptibility to MM development regarding *PD-1* gene polymorphisms also indicated a significant association with a high PD-1 expression haplotype [25], which is in opposition to the *CTLA-4* gene, as its lower expression level has been reported to be involved in MM development [26,27]. These results together with our findings might suggest that, among immune checkpoints, PD-1 rather than CTLA-4 could have potential as a target for therapeutic blockade in a subset of RRMM patients, if the relevant expression of T cells is observed.

Although a weakness of our study is that the relatively small size of the patient cohort inhibits our ability to make strong conclusions, noteworthy is its accordance with the results of two recent phase III clinical trials (namely KEYSTONE-183 and KEYSTONE-185 conducted in RR and ND MM patients, respectively) [17,18]. Both trials showed an unfavorable benefit-to-risk profile after administration of pembrolizumab (a PD-1 inhibitor) in combination with dexamethasone and immunomodulatory drugs (IMIDs). While these trials failed to show higher overall response rate (ORR) or TTP/progression-free-survival (PFS) in the experimental arms, they also demonstrated a much higher frequency and severity of immune-related adverse events (iRAEs) compared to those observed in a checkpoint blockade in other malignancies [17,18]. Moreover, regarding the clinical stages of MM, iRAEs were more pronounced in NDMM patients than in RRMM subjects, probably due to a less exhausted immune system at disease onset [19] and inappropriate prevention of autoimmunity, which clearly confirms the findings presented in our current work. In addition, we displayed a predictive value of the clinico-pathological characteristics of MM patients enrolled in the study, demonstrating the impact of β2-microglobulin, plasmacyte, and hemoglobin levels on the time to relapse, thus indicating the representativeness of our cohort of patients.

In the immunotherapy of MM patients, except pembrolizumab, several other ICIs have been administered, including nivolumab (anti-PD-1 mAb) and atezolizumab (anti-PD-L1 mAb) alone, or in combination with conventional chemotherapeutics and/or IMIDs, which are showing a disappointing clinical response in the majority of cases [23,55]. Available data showed that the low efficacy of conventional checkpoint inhibitors may be caused by the existence of compensatory inhibitory mechanisms related to the up-regulation of the other checkpoints, such as VISTA or TIM-3 [56,57]. Remarkably, our study clearly showed that an inappropriate level of PD-1 and CTLA-4 checkpoint expression in CD4 T cells in MM patients may also be responsible for the failure of ICIs, and in a proportion of NDMM patients, therapy with ICIs might even be deleterious by shortening the TTP. Our finding implies the significance of checkpoint level assessment in MM patients for predicting the clinical response rate to ICIs and for determining effective therapeutic strategies. Therefore, recently discovered and developed small-molecule inhibitors (SMIs) of checkpoint receptors provide an alternative and promising approach to the immunotherapy of cancers, and are of growing interest due to several desirable benefits they offer [58]. One of these benefits is the capability of SMIs to target more than one checkpoint protein, and the selectivity against other immune checkpoints and enzymes involved in the transcription of genes engaged in tumor suppression [58,59]. In consequence, SMIs reveal the potency for inducing a greater clinical response rate compared to conventional ICIs [58,59,60]. This pleiotropic activity of SMIs seems to be an attractive property for the area of MM immunotherapy aimed at breaking immune suppression, and appears to be of special interest, especially in view of our notion that PD-1 and CTLA-4 could be less accessible for ICIs in MM. Having ascertained that reversion of both the checkpoint levels and T cell reactivity observed in our cohort of RRMM patients might improve the clinical response to therapeutic inhibitors in terms of delayed relapse, the development of methods targeting the restoration of PD-1 expression in MM T cells, for example, SMIs modulating PD-1 gene transcription, seems to be desirable. In fact, epigenetic small-molecule modulators of PD-L1 and PD-L2 genes’ transcription have been shown to up-regulate the expression of these ligands, thus making them more amenable for effective inhibition of the PD-1/PD-L interaction when combined with a PD-1 blocking antibody in mice [61]. This is in accordance with reports showing that PD-L1 expression in cancers might predict a better response to ICIs and improve survival [62]. In cancer treatment, SMIs may act alone or in combination with approved therapies, namely chemo-, radio-, or immunotherapy, including monoclonal antibodies directed toward PD-1/PD-L1 or CTLA-4, such as nivolumab, pembrolizumab, atezolizumab, or ipilimumab (anti-CTLA-4 mAb) [63,64,65,66,67]. Several epigenetic SMIs have also recently been suggested as potent adjuvant agents for combined treatment in numerous types of advanced cancers [58]. Of these, entinostat, panobinostat, and azacitidine are currently in clinical trials in combined therapy with pembrolizumab or ipilimumab in immunogenic cancer patients [63,66,67]. Encouraging results in pre-clinical studies on PD-1-derived CA170 (a small molecule dually targeting PD-L1 and VISTA pathways) reporting high efficacy in the suppression of tumor growth at well-tolerated doses has prompted the advancement of CA170 to clinical trials [59].

In conclusion, the results of our study together with the impressive clinical response to the checkpoint blockade seen in patients with solid tumors, expressing regulatory receptors at a relevant level [16], strengthens the suggestion of a predictive role of checkpoint expression in this therapeutic approach in MM. Therefore, considering novel therapies and potent combinations, estimation of the immune checkpoint levels in T cells before the administration of inhibitors in the different clinical stages of MM is warranted for excluding patients with checkpoint suboptimal levels in order to avoid very limited effectiveness of the anti-myeloma response, early relapse, and/or severe aggravation of autoimmune adverse events.

## 4. Materials and Methods

### 4.1. Samples

The study group of patients studied consisted of a total of 40 active myeloma patients (26 newly diagnosed (NDMM) and 14 relapsed/refractory (RRMM)). Patients were recruited in two centers: the Department of Hematology and Bone Marrow Transplantation at Wroclaw Medical University and the Department of Hematological Oncology at the Provincial Hospital in Opole. The diagnosis of MM was based on the criteria set out by the International Myeloma Working Group (IMWG) [68]. The disease stage was determined according to the International Staging System (ISS) upon entry into the study [69]. RRMM patients were treated with chemotherapy, IMIDs, and a proteasome inhibitor; no patient enrolled in the study received prior treatment with stem cell transplantation (SCT) or ICIs.

The control population comprised 20 healthy individuals matched for age and sex; they had been without any treatment affecting the immune system for six months before entering the study. Patients with a simultaneous active or chronic infection, diabetes, autoimmune disease, or with a history of other malignancies or connective tissue diseases were excluded from the study. Blood samples from all participants were collected after informed consent in accordance with the Declaration of Helsinki and approval from the Institutional Local Research Bioethics Committee at Wroclaw Medical University.

### 4.2. Clinical and Laboratory Characteristics of Patients

The main characteristics of MM patients are summarized in Table S1. Of the 26 NDMM patients, the majority were women (n = 17, 65.4% vs. n = 9, 34.6%). The median age at diagnosis was 66.0 years old (range 50–76). According to the ISS, 5 patients (19.0%) were classified as stage I, 10 (38.5%) were classified as stage II, and 11 (42.5%) were classified as stage III. The immunoglobulin subtype was IgG for 18 patients (69.0%), and IgA for 3 patients (11.5%). Five patients (19.5%) had light chain only. Of the NDMM patients, the majority (n = 16, 61.5%) had light chain kappa, 9 patients (34.5%) had light chain lambda. A majority of the NDMM patients presented with osteolytic bone lesions (n = 15, 60.0%), had a serum β2-microglobulin level ≥ 3.5 mg/L (n = 18, 69.0%), and a hemoglobin value ≤ 12 g/dL (n = 23, 88.5%). A creatinine level ≥ 2.0 mg/dL and a serum calcium value ≥ 10 mg/dL were present in 9 (34.5%) NDMM patients. An LDH level higher than 190 U/L and a platelet value less than 100,000/mm^3^ was present in 6 (23.0%) and 2 (7.5%) patients, respectively.

Of the 14 RRMM patients, 10 were men (71.4%) and 4 were women (28.6%). The median age at diagnosis was 72.0 years old (range 65–75). According to the ISS, 1 patient (7.0%) was classified as stage I, 6 (43.0%) were classified as stage II, and 7 (50.0%) were classified as stage III. The immunoglobulin subtype was IgG for 9 patients (64.0%), and IgA for 3 patients (21.5%). Two patients had light chain only (14.5%). Seven RRMM patients had light chain kappa (50.0%) and 7 had light chain lambda. The majority of the RRMM patients (n = 13, 92.8%) presented with osteolytic bone lesions. Of the RRMM patients, a majority had a serum β2-microglobulin level ≥ 3.5 mg/L (n = 9, 64.0%), a serum calcium level ≥ 10 mg/dL (n = 11, 78.5%), and a hemoglobin value ≤ 12 g/dL (n = 8, 57.0%). Four RRMM patients (28.5%) had a creatinine level ≥ 2.0 mg/dL. An LDH level higher than 190 U/L and a platelet value less than 100,000/mm^3^ was present in 2 (14.0%) patients and 1 (7.0%) patient, respectively. Of the RRMM patients, 12 subjects (85.5%) received bortezomib (BTZ)-based therapy, and 11 patients (78.5%) were treated with IMiDs. The majority of RRMM patients (n = 8, 57.0%) received 1–3 therapy lines, while the remainder (n = 6, 43.0%) received ≥ 4 therapy lines.

### 4.3. Isolation of Peripheral Blood Mononuclear Cells (PBMCs)

Peripheral venous blood from MM patients and healthy individuals was collected in tubes containing lithium heparin as anticoagulant. Peripheral blood mononuclear cells (PBMCs) were isolated by buoyant density-gradient centrifugation on Lymphoflot (Bio-Rad Medical Diagnostics GmbH, Dreieich, Germany). After centrifugation, the PBMCs were washed three times in sterile phosphate-buffered saline (PBS) (without Ca^2+^ and Mg^2+^), and then suspended in 95% fetal calf serum (CytoGen GmbH, Sinn, Germany) containing 5% DMSO (Sigma-Aldrich, St. Gallen, Switzerland) and cryopreserved prior to use.

### 4.4. Determination of Immune Checkpoints (PD-1 and CTLA-4), CD28 and CD69 Expression

Multicolor flow cytometry was used to analyze the PBMCs of MM patients and healthy controls for the expression of PD-1 and CTLA-4 proteins in pooled CD4^+^ T cells as well as their subsets: Treg cells (CD4^+^CD127^−^ cells) and Teff cells (CD4^+^CD127^+^ cells). The expression of CD28 and CD69 molecules was detected on gated CD4^+^ T cells. According to standard protocols, isolated PBMCs were stained with several combinations of fluorochrome-conjugated monoclonal primary antibodies (mAbs) purchased from Pharmingen (Pharmingen, BD Biosciences, San Diego, CA, USA): FITC anti-human CD3 (Catalog #555332), FITC anti-human CD127 (Catalog # 560549), PerCP anti-human CD4 (Catalog #566924), PE anti-human PD-1 (Catalog #560795), PE anti-human CTLA-4 (Catalog #555853), PE anti-human CD69 (Catalog #555531), and PE anti-human CD28 (Catalog #555729). Appropriate fluorochrome-labeled isotype control antibodies included from Pharmingen (Pharmingen, BD Biosciences, San Diego, CA, USA) were used to confirm expression specificity and for gate settings in each case (FITC mouse IgG1: Catalog #555748, PE mouse IgG1: Catalog #554680, PE mouse IgG2a: Catalog #555574, PE mouse IgG1: Catalog #555749). Briefly, refrozen PBMCs were washed twice in PBS, divided into tubes at a concentration of 5 × 10^5^ cells per tube, and incubated with appropriate antibodies for 30 min at 4 °C in the dark. Excess unbound antibodies were removed by two washes with PBS. Following washing, the cells were fixed in PBS containing 1.5% paraformaldehyde (Catalog #P6148, Sigma-Aldrich, St. Gallen, Switzerland) and analyzed by flow cytometry. Finally, a total of 100,000 events per sample were acquired using a FACSCalibur flow cytometer (Becton-Dickinson, BD Biosciences, San Diego, CA, USA) equipped with Cell Quest software 3.3 (Becton-Dickinson, BD Biosciences, San Diego, CA, USA). Data were analyzed by Cell Quest software and the results were expressed as the proportions of CD3^+^CD4^+^ (CD4^+^ T cells) as well as CD4^+^CD127^−^ (Treg) and CD4^+^CD127^+^ (Teff) cells co-expressing PD-1 or CTLA-4. The percentages of CD4^+^ T cells co-expressing CD69 and the frequencies of CD4^+^CD28^−^ T cells were also examined. The gating strategy is presented in Figure S1 in supplementary materials. In order to demonstrate quantitative expression of immune checkpoints as well as CD69 protein at the single-cell levels, the results are shown as mean fluorescence intensity (MFI) values and expressed in arbitrary units (AU).

### 4.5. Statistical Analysis

Statistical analyses of the clinical data and laboratory findings were performed using the Statistica 7.1 package (TIBCO Software Inc., Palo Alto, CA, USA) and GraphPad Prism 8.01 (GraphPad Software, San Diego, CA, USA). Clinical parameters were presented as absolute numbers and percentages for frequencies. For all other analyzed variables, the median values and 25th and 75th interquartile ranges (IQ ranges) were calculated. As collected data were not normally distributed and/or had heterogeneous variances, differences between examined groups were evaluated using a one-way analysis of variance (ANOVA), with the Kruskal–Wallis or the Mann–Whitney U test as nonparametric alternatives. The Kaplan–Meier method was used to plot survival curves and the difference between curves was calculated by the log-rank test. A *p* value ≤ 0.05 was considered significant.

## Data Availability

All data generated or analyzed during this study are available on request from the corresponding author.

## Supplementary Materials

The following supporting information can be downloaded at: https://www.mdpi.com/article/10.3390/ijms24065730/s1.
