# Supplementary material for: Inappropriate Expression of PD-1 and CTLA-4 Checkpoints in Myeloma Patients Is More Pronounced at Diagnosis: Implications for Time to Progression and Response to Therapeutic Checkpoint Inhibitors
Source: Int J Mol Sci. 2023 Mar 17;24(6):5730. doi: 10.3390/ijms24065730 (PMC10056286; doi:10.3390/ijms24065730)
Supplement: Supplementary file 1 [file ijms-24-05730-s001.zip › ijms-2100416-supplementary.pdf]

Table S1. Patient clinical and laboratory characteristics.

Parameters	Newly Diagnosed (NDMM)	Relapsed/Refractory (RRMM)	Total
Number of patients, n (%)	26 (65%)	14 (35%)	40 (100%)
Age of patients, years			
median (range)	66 (50-76)	72 (65-75)	69 (59-76)
Gender , n (%)			
Female	17 (65.38%)	4 (28.57%)	21 (52.5%)
Male	9 (34.62%)	10 (71.43%)	19 (47.5%)
ISS stage at diagnosis, n (%)			
I	5 (19.23%)	1 (7.14%)	6 (15.00%)
II	10 (38.46%)	6 (42.86%)	16 (40.00%)
III	11 (42.31%)	7 (50.00%)	18 (45.00%)
Type of myeloma, n (%)			
IgG	18 (69.23%)	9 (64.28%)	27 (67.50%)
IgA	3 (11.54%)	3 (21.43%)	6 (15.00%)
Light chain disease	5 (19.23%)	2 (14.29%)	7 (17.50%)
Type of Ig light chain (serum), n (%)			
Kappa	16 (61.54%)	7 (50.00%)	23 (57.50%)
Lambda	9 (34.62%)	7 (50.00%)	16 (40.00%)
Unknown	1 (3.84%)	0 (0.00%)	1 (2.50%)
Osteolytic bone lesion/s, n (%)			
Present	15 (57.69%)	13 (92.86%)	28 (70.00%)
Absent	11 (42.31%)	1 (7.14%)	12 (30.00%)
Morphological indicators, n (%)			
Hemoglobin ≤ 12 g/dL	23 (88.47%)	8 (57.14%)	31 (77.50%)
Platelets < 100,000/mm^3	2 (7.69%)	1 (7.14%)	3 (7.50%)
Biochemical indicators, n (%)			
β-2 microglobulin ≥ 3.5 mg/L	18 (69.23%)	9 (64.29%)	27 (67.50%)
Creatinine ≥ 2.0 mg/dL	9 (34.62%)	4 (28.57%)	13 (32.50%)
LDH > 190 U/L	6 (23.08%)	2 (14.29%)	8 (20.00%)
Serum calcium ≥ 10 mg/dL	9 (34.62%)	11 (78.57%)	20 (50.00%)
Prior treatment, n (%)			
1-3 therapy lines	0 (0%)	8 (57.14%)	8 (20.00%)
≥ 4 therapy lines	0 (0%)	6 (42.86%)	6 (15.00%)
BTZ based therapy	0 (0%)	12 (85.71%)	12 (30.00%)
IMiD therapy	0 (0%)	11 (78.57%)	11 (27.50%)
No therapy	26 (100%)	0 (0%)	26 (65.00%)

Abbreviations: BTZ, bortezomib; LDH, lactate dehydrogenases; ISS, International Staging System; IMiD, immunomodulatory drug.

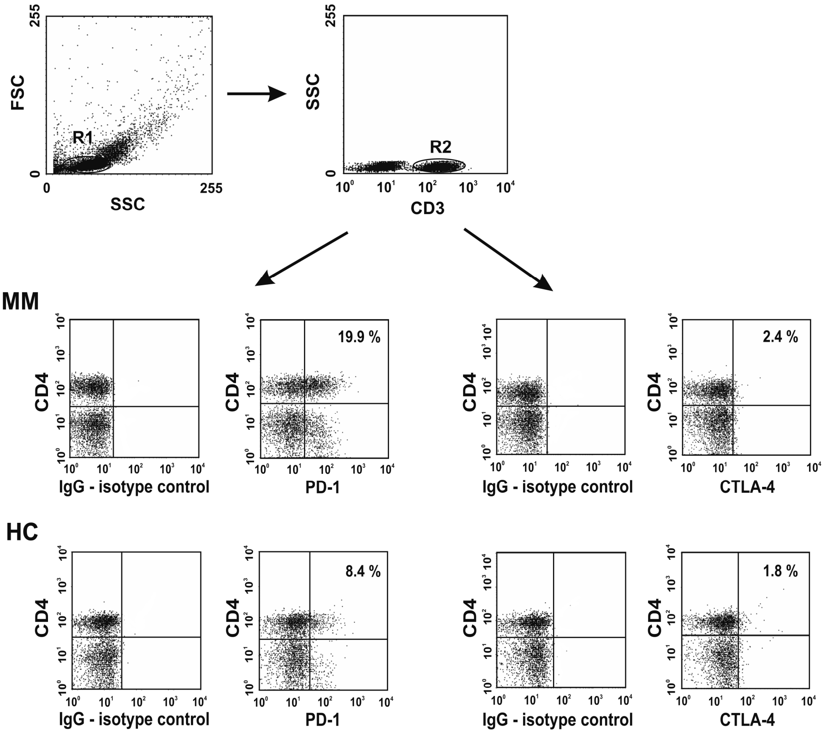

(a)

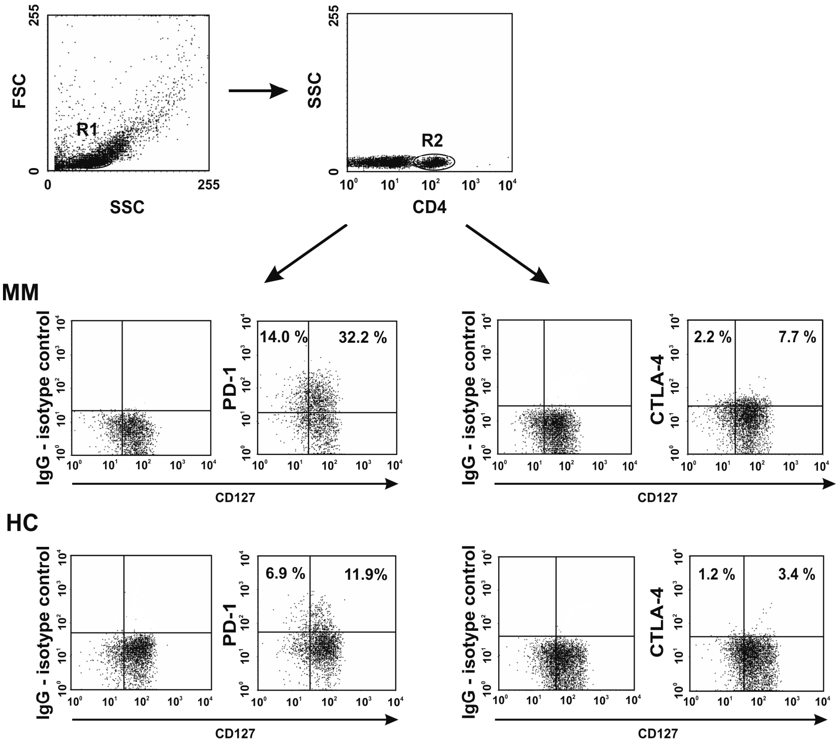

(b)

Figure S1. Representative dot plots from MM patient and healthy control (HC). Dot plots demonstrate the gating strategy for assessing the proportion of CD4^{+} T cells (CD3^{+}CD4^{+}), Treg (CD4^{+}CD127^{-}) and Teff (CD4^{+}CD127^{+}) cells co-expressing PD-1 or CTLA-4 protein. (**a**) Dot plots show the method for analyzing PD-1 or CTLA-4 positive CD4^{+} T cells. Lymphocytes were gated (R1) based on their FSC/SSC properties, and then T cells were identified on the SSC/CD3 profile (R2). The R2 gated events were next analyzed for CD4 and PD-1 or CTLA-4 staining. Numbers on dot plots show the percentage of PD-1 or CTLA-4 positive CD4^{+} T cells. (**b**) Dot plots show the method for analyzing PD-1 or CTLA-4 positive Treg and Teff cells. The R1 gated lymphocytes were subsequent gated on SSC/CD4 (R2) to identify CD4^{+} cells. The R2 gated populations were then analyzed for CD127 and PD-1 or CTLA-4 staining. Numbers on dot plots show the percentage of PD-1 or CTLA-4 positive Treg and Teff cells.
